# Paternal SIRT1 Inhibition During Capacitation Imprints Histone 4 Hyperacetylation in Mouse Zygotes and Blastocysts

**DOI:** 10.1111/rda.70291

**Published:** 2026-09-30

**Authors:** M. Iniesta‐Cuerda, C. Vidal‐Garcia, J. Nevoral

**Affiliations:** ^1^ Biomedical Center, Faculty of Medicine in Pilsen Charles University Pilsen Czech Republic; ^2^ Grupo SaBio, IREC (UCLM‐CSIC‐JCCM), ETSIAMB Albacete Spain; ^3^ Department of Histology and Embryology, Faculty of Medicine in Pilsen Charles University Pilsen Czech Republic

**Keywords:** acH4K16, paternal imprinting, SIRT1

## Abstract

Sirtuin 1 (SIRT1) is a NAD^+^‐dependent deacetylase involved in chromatin remodelling. In this study, we evaluated whether sperm SIRT1 inhibition affects early embryonic chromatin dynamics. Sperm were treated with sirtinol during capacitation or during both capacitation and fertilization. SIRT1 inhibition increased acH4K16 in the male pronucleus without affecting SIRT1 levels, whereas the female pronucleus remained largely unchanged, except for reduced SIRT1 when inhibition was extended to fertilization. Notably, embryos derived from treated sperm maintained elevated acH4K16 at the blastocyst stage. These findings indicate that sperm SIRT1 modulation can influence paternal chromatin remodelling and early embryonic epigenetic regulation.

## 1 Introduction

Sirtuin 1 (SIRT1) is a NAD^+^‐dependent deacetylase widely recognized for its role in regulating chromatin structure, transcriptional activity and cellular stress responses. In the context of reproduction, SIRT1 has been implicated in multiple processes related to testicular physiology, gamete function and early embryonic development, including spermatogenesis efficiency/sperm capacitation (Iniesta‐Cuerda et al. 2026), the maternal‐to‐embryonic transition (Nevoral et al. 2024) and epigenetic remodelling (Adamkova et al. 2017). In spermatozoa, where transcription is largely inactive, post‐translational modifications of proteins and chromatin represent key regulatory mechanisms that modulate functional competence. Among these, acetylation has emerged as a key molecular switch during sperm capacitation, influencing the acquisition of fertilizing ability (Ritagliati et al. 2018). Moreover, histone acetylation promotes a more open chromatin configuration associated with transcriptional competence, a feature significantly observed during early embryonic genome activation (EGA; Adenot et al. 1997). A previous unpublished study has demonstrated that pharmacological inhibition of SIRT1 during sperm capacitation can improve fertilization outcomes, likely through enhanced acetylation‐dependent remodelling processes that facilitate sperm functional activation (Iniesta‐Cuerda et al. 2026, under revision). Consistent with this, SIRT1 inhibition has also been associated with increased histone H4 acetylation at lysine 16 (acH4K16) levels in blastocysts, suggesting that its effects may extend beyond sperm physiology and influence early embryonic chromatin regulation (Nevoral et al. 2024).

Increasing evidence indicates that paternal factors delivered by the spermatozoon can influence early embryonic development through epigenetic mechanisms. Shortly after fertilization, the zygote undergoes extensive chromatin reorganization and epigenetic reprogramming, particularly within the paternal genome, which must rapidly transition from a highly compacted sperm chromatin state to a transcriptionally permissive configuration (Balhorn 2007). This process involves dynamic changes in histone modifications, including acetylation marks that facilitate chromatin relaxation and genome activation (Adenot et al. 1997). However, whether modulation of sperm SIRT1 activity prior to fertilization can influence these early paternal chromatin remodelling events remains largely unexplored. In this context, we investigated whether inhibition of sperm SIRT1 during capacitation, either alone or maintained during fertilization, affects SIRT1 distribution and acH4K16 patterns in the resulting zygotes and derived blastocysts, with particular attention to potential paternal‐specific epigenetic effects.

## 2 Materials and Methods

### 2.1 Animals and Ethics

All animal procedures complied with Act No. 246/1992 on the Protection of Animals against Cruelty and were approved by the Animal Welfare Advisory Committee at Charles University, Faculty of Medicine in Pilsen, and the Ministry of Education, Youth and Sports of the Czech Republic (Approval No. MSMT‐33798/2021‐4). C57BL/6N mice were maintained under standard conditions with *ad libitum* access to food and water.

### 2.2 Sperm Capacitation and SIRT1 Inhibition

Spermatozoa were collected from the cauda epididymides of adult males (14‐ to 20‐week‐old) and capacitated in modified human tubal fluid supplemented with 0.4% BSA (mHTF) for 1 h at 37°C under 5% CO_2_. SIRT1 activity was inhibited using 10 μM sirtinol (Abcam, AB141263), based on our previous work (Iniesta Cuerda et al., manuscript under revision), added either during sperm capacitation alone or throughout both capacitation and sperm‐oocyte co‐incubation during fertilization (referred to as Sirtinol in the figures). Control sperm were treated with the corresponding vehicle (DMSO; Vehicle) at the same volume used in the treated samples, maintaining a final concentration below 0.1% (v/v).

### 2.3 In Vitro Fertilization and Embryo Culture

For in vivo ovulated mature oocytes 8‐ to 12‐week‐old female mice were used, PMSG was followed by hCG 48 h later, and ovulated cumulus‐oocyte complexes were collected from the oviduct 15–16 h post‐hCG. Cumulus‐oocyte complexes were coincubated with capacitated sperm in mHTF for 5.5 h. In the continuous inhibition design, 10 μM sirtinol was also included in the fertilization medium. Presumed zygotes were either fixed for immunocytochemistry or cultured in KSOM supplemented with 0.1% (w/v) BSA for up to 4 days. Maternal and paternal pronuclei were distinguished based on size, with the paternal pronucleus consistently larger than the maternal one (Adenot et al. 1997).

### 2.4 Immunocytochemistry and Image Analysis

Zygotes and blastocysts were fixed in 4% paraformaldehyde, permeabilized (PBS‐0.02% Tween‐0.1% Triton X‐100), and blocked in PBS with 5% BSA. Samples were incubated with primary antibodies against anti‐mouse‐SIRT1 and anti‐rabbit‐acH4K16 (1:200; ab110304 and ab109463, respectively; Abcam, UK), followed by antimouse‐Alexa‐Fluor‐488‐ and anti‐rabbit‐647‐conjugated secondary antibodies. Nuclei were counterstained with DAPI. Images were acquired using a spinning‐disk confocal microscope (Olympus IX83), and fluorescence intensity was quantified with ImageJ.

### 2.5 Statistical Analysis

Data normality was assessed using the Shapiro–Wilk test. Depending on the data distribution, comparisons between groups were performed using either Student's *t*‐test or the Mann–Whitney *U* test. Only acH4K16 levels in the male 2PN following SIRT1 inhibition during capacitation, and SIRT1 and acH4K16 levels in blastocysts from the co‐incubation inhibition group were normally distributed. Statistical analyses were performed using GraphPad Prism software. Data are presented as mean ± SEM, and differences were considered statistically significant at *p* < 0.05.

## 3 Results

### 3.1 Sperm SIRT1 Inhibition During Capacitation Induces Paternal‐Specific H4 Hyperacetylation in Zygotes and Blastocysts

To assess whether sperm SIRT1 modulation affects early post‐fertilization chromatin remodelling, spermatozoa were treated with sirtinol during capacitation prior to IVF. Immunocytochemical analysis confirmed the presence of SIRT1 in mouse sperm, supporting the biological relevance of pharmacological inhibition (Figure 1A). Subsequent immunofluorescence analysis of the resulting zygotes revealed a pronounced increase in acH4K16 in the male pronucleus compared with controls (*p* < 0.05; Figure 1B). SIRT1 signal remained unchanged in both pronuclei, indicating that hyperacetylation reflects functional inhibition rather than altered protein abundance (Figure 1C). To assess whether this epigenetic effect persisted during preimplantation development, embryos derived from treated sperm were cultured to the blastocyst stage. Blastocysts originating from sperm exposed to SIRT1 inhibition during capacitation exhibited sustained elevations in H4K16ac levels compared with controls (*p* > 0.05; Figure 1E). However, SIRT1 expression levels in blastocysts were not significantly affected under this experimental design (Figure 1F).

**FIGURE 1:**
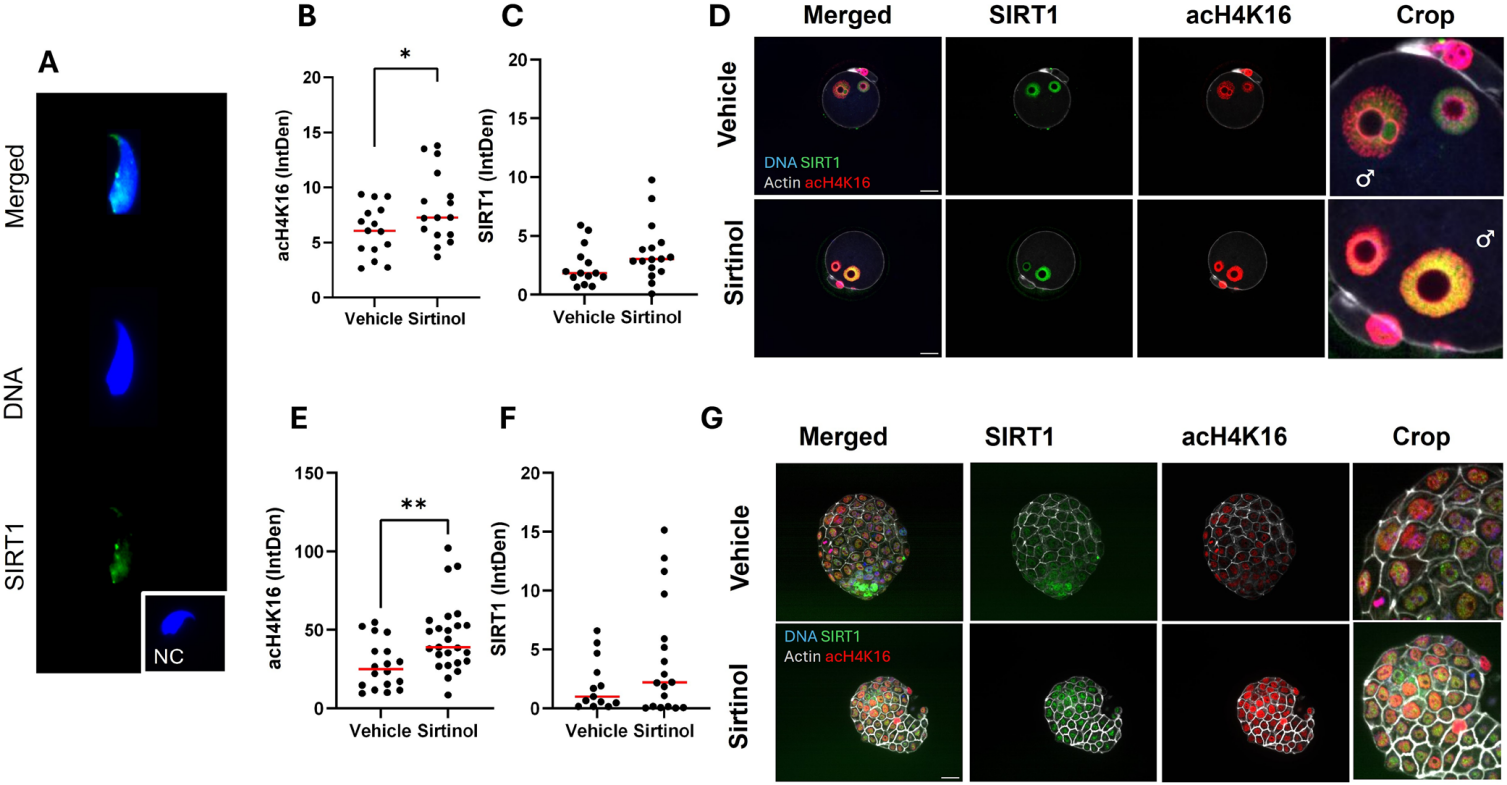
(A) SIRT1 is present in the sperm head. Its inhibition during sperm capacitation increases acH4K16 in the (B) male pronucleus (C) without altering SIRT1. At the blastocyst stage, acH4K16 is similarly increased (E), whereas SIRT1 remains unchanged (F). (D, G) Representative images. Integrated density (IntDen) of the fluorescence signal is shown.

### 3.2 Extending SIRT1 Inhibition to Fertilization Alters Female Pronuclear SIRT1 and Maintains H4 Hyperacetylation

We next evaluated whether extending SIRT1 inhibition to the fertilization period would further influence early embryonic chromatin remodelling. When sirtinol was present during both sperm capacitation and sperm‐oocyte co‐incubation, zygotes again displayed increased acH4K16 specifically in the male pronucleus but not in the female pronucleus (*p* < 0.05; Figure 2A). Under this condition, however, a reduction in SIRT1 signal was detected in the female pronucleus (*p* < 0.05), whereas the male pronucleus remained unchanged (*p* > 0.05; Figure 2B). Embryos derived from this experimental design retained elevated acH4K16 at the blastocyst stage (*p* < 0.05; Figure 2D), similar to the capacitation‐only treatment. In contrast, SIRT1 expression in blastocysts was increased (*p* < 0.05; Figure 2E), suggesting that prolonged SIRT1 inhibition may additionally influence embryonic SIRT1 regulation during preimplantation development.

**FIGURE 2:**
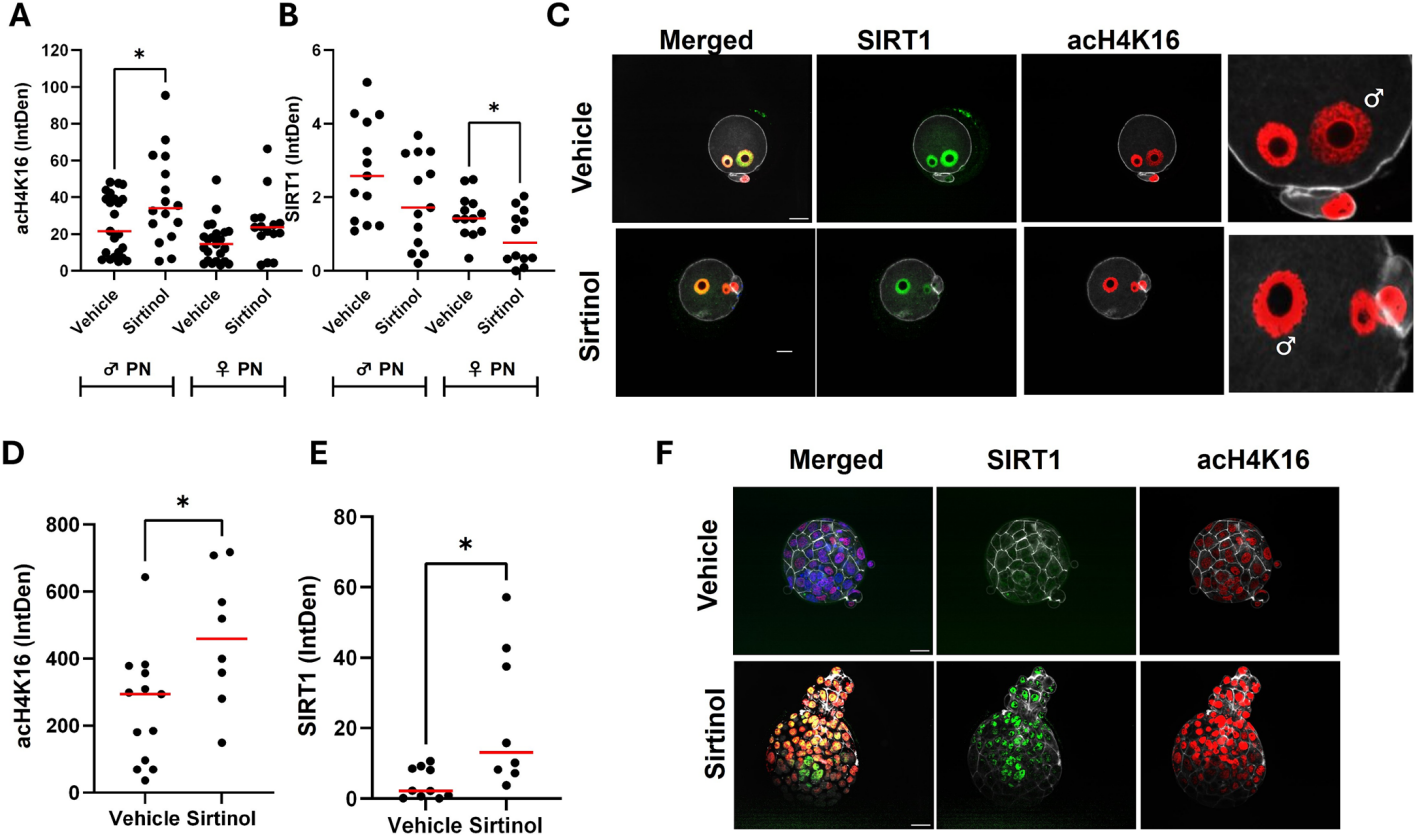
SIRT1 inhibition during capacitation and fertilization increases acH4K16 in the (A) male pronucleus (B) without altering SIRT1, whereas decreasing it in the female pronucleus. At the blastocyst stage, both (D) acH4K16 and (E) SIRT1 are increased. (C, F) Representative images. Integrated density (IntDen) of the fluorescence signal is shown.

## 4 Discussion

In spermatozoa, where transcriptional activity is largely absent, the functional consequences of SIRT1 activity remain unclear but may involve post‐translational regulation of proteins and chromatin‐associated factors. Previous work has shown that pharmacological inhibition of SIRT1 during sperm capacitation improves fertilization outcomes, likely by favouring acetylation‐dependent processes that facilitate sperm functional activation (Iniesta‐Cuerda et al. 2026). The present results extend these observations by demonstrating that sperm SIRT1 inhibition also leaves an epigenetic footprint after fertilization, manifested as selective elevation of acH4K16 in the male pronucleus.

A striking feature of our findings is the strict paternal specificity of this effect. In both experimental models, increased acH4K16 was observed exclusively in the male pronucleus, whereas the female pronucleus remained unaffected. Shortly after fertilization, the paternal genome undergoes extensive chromatin remodelling, during which protamines are rapidly replaced by histones derived primarily from the oocyte. One possible explanation for the observed pattern is that histones incorporated into the paternal chromatin already carry acetylation marks when deposited into the male pronucleus (Adenot et al. 1997; Balhorn 2007). However, since SIRT1 inhibition was restricted to sperm prior to fertilization and oocytes were never exposed to the inhibitor, this possibility appears unlikely. Moreover, if the acetylated histones detected in the paternal pronucleus were mainly derived from the oocyte histone pool, a similar pattern would be expected in the female pronucleus. The absence of such a pattern suggests that the increased acetylation observed in the paternal pronucleus is unlikely to result from alterations in the oocyte‐derived histone reservoir.

Importantly, SIRT1 levels in the male pronucleus were not altered by the treatments, and basal pronuclear SIRT1 abundance was comparable, indicating that the observed hyperacetylation cannot be explained by differences in SIRT1 expression. Rather, these findings support the interpretation that the increase in acH4K16 results from functional inhibition of sperm SIRT1 prior to fertilization. Notably, no compensatory effect exerted by the oocyte machinery was apparent, despite the extensive chromatin remodelling that normally accompanies fertilization (Adenot et al. 1997; Balhorn 2007). Consistent with this interpretation, embryos derived from SIRT1‐inhibited sperm retained elevated acH4K16 levels at the blastocyst stage in both experimental settings, suggesting that this epigenetic alteration is not readily erased during preimplantation development. Interestingly, in this experimental design we previously observed that embryonic development from the two‐cell stage onwards was improved (Iniesta‐Cuerda et al. 2026), indicating that modulation of sperm SIRT1 activity may influence early developmental progression. Taken together, these observations should be regarded as preliminary. Further studies will be required to better understand the potential consequences of sperm SIRT1 modulation prior to fertilization and its possible implications for early embryonic development.

## Author Contributions

Conceptualization: María Iniesta‐Cuerda and Jan Nevoral; methodology: María Iniesta‐Cuerda, Jan Nevoral and Clara Vidal‐Garcia; investigation: María Iniesta‐Cuerda and Jan Nevoral; writing – original draft preparation: María Iniesta‐Cuerda; writing‐review and editing: Jan Nevoral. All authors have read and agreed to the published version of the manuscript.

## Funding

This work was supported by the Ministry of Education, Youth and Sports—Czech Republic (Crossref Funder ID: 501100001823; ROR ID: 037n8p820): the Cooperatio Programme, research areas MED/DIAG and MED/IMMU of Charles University; Project No. SVV 260 773; European Regional Development Fund—Projet ‘Fighting INfectious Diseases’ (no. CZ.02.1.01/0.0/0.0/16_019/0000787) and the Technology Agency of the Czech Republic (grant no. QL24010123). Additional Funding was provided by the Regional Government of Castilla‐La Mancha (Spain; Project SBPLY‐23‐180225‐000184).

## Conflicts of Interest

The authors declare no conflicts of interest.

## Data Availability

The data that support the findings of this study are available from the corresponding author upon reasonable request.
